# Breakfast Nutritional Quality and Cognitive Interference in University Students from Mexico City

**DOI:** 10.3390/ijerph16152671

**Published:** 2019-07-26

**Authors:** Reyna Sámano, Carmen Hernández-Chávez, Gabriela Chico-Barba, Armando Córdova-Barrios, Mayela Morales-del-Olmo, Hortensia Sordo-Figuero, Miguel Hernández, Carmen Merino-Palacios, Lucero Cervantes-Zamora, Hugo Martínez-Rojano

**Affiliations:** 1 Departamento de Nutrición y Bioprogramación, Instituto Nacional de Perinatología, Secretaría de Salud, Montes Urales 800, Miguel Hidalgo, Lomas Virreyes, Ciudad de México CP 11000, México; 2 Departamento de Neurobiología del Desarrollo, Instituto Nacional de Perinatología, Secretaría de Salud, Montes Urales 800, Miguel Hidalgo, Lomas Virreyes, Ciudad de México CP 11000, México; 3 Escuela de Enfermería, Universidad Panamericana, Augusto Rodin 498, Insurgentes Mixcoac, Alcaldía Benito Juárez CP 03920, México; 4 Coordinación de Psicología, Instituto Nacional de Perinatología, Secretaría de Salud, Montes Urales 800, Miguel Hidalgo, Lomas Virreyes, Ciudad de México CP 11000, México; 5 Universidad del Valle de México-Chapultepec, Avenida Observatorio 400, 16 de Septiembre, Ciudad de México CP 11810, México; 6 Departamento de Posgrado e Investigación, Escuela Superior de Medicina del Instituto Politécnico Nacional, Plan de San Luis y Díaz Mirón s/n, Colonia Casco de Santo Tomas, Alcaldía Miguel Hidalgo, Ciudad de México CP 11340, México; 7 Coordinación de Medicina Laboral, Instituto de Diagnóstico y Referencia Epidemiológicos, Secretaría de Salud, Francisco de P, Miranda 177, col. Unidad Lomas de Plateros Alcaldía Álvaro Obregón C.P, Ciudad de México 01480, México

**Keywords:** cognitive interference, breakfast, Stroop, young adults, university students

## Abstract

Skipping breakfast might have an impact on cognitive functions, such as interference, which is a basic capacity of executive functions that denotes the possibility of controlling an automated response. This study aimed to analyze the association between nutritional quality of breakfast and cognitive interference in a sample of university students. A cross-sectional study was conducted, a total of 422 students between 18 and 25 years participated. Cognitive interference was assessed with the Stroop Test. Breakfast was assessed with a questionnaire assigning a score for each serving of each food group that was consumed. Logistic regression models were performed. The performance in cognitive tasks was slower in those who had a poor breakfast (32.9 ± 6 vs 29.3 ± 6 s, *p* < 0.050). Poor cognitive interference was greater in students with poor breakfast (53% versus 23%, *p* = 0.001). A slower word reading was associated with skipping vegetables (OR: 2.78, 95% CI: 0.09–2.13), and cereals (OR: 1.70, 95% CI: 1.03–2.81). Wrong color identification was associated with skipping fruits (OR: 1.55, 95% CI: 1.43–1.99) and animal protein sources (OR: 1.63, 95% CI: 1.07–2.49). Skipping fat-rich cereals was a protector factor (OR: 0.55, 95% CI: 0.36–0.85). Difficulty in the ability to inhibit interference was associated with skipping vegetables (OR: 2.72, 95% CI: 1.25–4.80) and cereals (OR: 2.65, 95% CI: 1.28–4.68). The nutritional quality of breakfast was associated with the time spent answering the Stroop test, but not with cognitive interference.

## 1. Introduction

Breakfast is the first and one of the most important meals of the day [1,2]. The consumption of breakfast in the younger population is less than 20% [3]; this prevalence tends to decrease as adolescents get older [4]. At the university level, one out of three students skip breakfast [5]; this omission entails diet consumption failures that are not compensated for in the rest of the meals and furthermore, can have an effect on the students’ performance [6]. Adequate breakfast habits are related to optimal physiological, psychological, and social health [7].

Breakfast with an adequate nutritional quality may improve cognitive performance and also regulate the quantity of food consumption in the following meals. A quality breakfast should provide energy (about 25% of the total energy intake of the day) and nutrients according to daily needs, and has to be accessible and affordable [1,7]. This may help prevent chronic diseases like overweight, obesity, and diabetes [8], and others such as eating disorders, particularly binges [9].

It has been found that an adequate breakfast improves the cognitive abilities and physical performance [10] and is associated to an adequate body mass index (BMI) [11]. Difficult tasks require increased energy expenditure in the brain [12]. It has been repeatedly suggested that breakfast has a direct effect on psychological functions [13,14,15,16], particularly in tasks that require memory, concentration, learning, and decision-making based on the available information [14,16].

Cognitive interference is the ability to inhibit or control impulsive or automatic responses and generate responses mediated by attention that are more adapted to the situation. This cognitive ability is part of executive functioning and contributes to anticipation, planning, and goal setting. Some examples are when we do not say something that we think is better not to say, or we chose to remain seated while attending class, studying at home, or working in the office despite the desire we have to get up. In this way, a good inhibition can favor better behaviors and notes in academic areas, more efficiency at work, and better personal relationships [17,18].

Interference inhibition is necessary for controlling selective attention; this neurological process is based on the dorsolateral prefrontal cortex. If the working memory has an increased load, attention can be lost and non-relevant information is more frequent.

A tool to measure cognitive interference is the Stroop test, which gives information about cognitive processes, such as selective attention, focused attention, response inhibition, interference control, and information processing speed [19,20]. The original Stroop test and all current variations consist of three conditions: A word task, a color task, and a color-word task. Based on the scores and times to response, the test was validated for Mexican population (Cronbach’s alpha = 0.767) [21].

Breakfast stops the depletion of nutrients that the central nervous systems needs for efficient cognitive functions [16]. The regular consumption of breakfast was associated with a better general performance throughout the day, but there remains the need to explore this relationship [13]. At breakfast, the consumption of foods like cereals, dairy products, and fruits is linked to benefits in weight, cholesterol level, digestive function, less cognitive bias, and less working accidents [22]. However, some studies did not find an association between cognitive interference and the daily consumption of breakfast [23,24]. The reason may be that several factors could affect cognitive interference, for instance; age, socioeconomic level, physiological status, and occupation [24]. Since there is not yet enough agreement, the aim of this study was to analyze the association between nutritional quality of breakfast and cognitive interference in a university student sample from Mexico City. Our hypothesis was that if the participants had a good nutritional quality breakfast, then they would have a good performance in the cognitive interference test.

## 2. Materials and Methods

This was a cross-sectional study including healthy young university students in Mexico City. It was a convenience sample, based on consecutive cases that compiled the following inclusion criteria: 18–25 years old, both sexes, clinically healthy, signed informed consent, and being a student of any of the bachelor programs in the university. Sample size was calculated based on the difference between two proportions; since the evidence is inconsistent about the effect of breakfast on cognitive interference, we expected a difference of 10% on good performance between breakfast groups (good quality versus poor quality); using a power of 80% and a confidence level of 95%, the total sample required was 356 subjects. Nevertheless, 15% was added for expected losses (due to incomplete questionnaires), so the total sample needed for the study was 419. A total of 460 participants were enrolled in the study; however, 38 were excluded because of incomplete questionnaires, giving a total sample of 422 participants. Most of the participants were from the middle-income level. The guidelines of the Helsinki Declaration were followed [25]. Participants were previously informed about the aim and assessments of the study. The questionnaire content and assessments did not involve any bio-psycho-social risk. This research had a registration number 212250-49541 by the Institutional Committees from the Instituto Nacional de Perinatología.

### 2.1. Instruments and Procedures

#### 2.1.1. Breakfast Nutritional Quality

A questionnaire was applied to obtain data about breakfast characteristics and general information about the participants, like age, sex, and the grade point average in their last semester. The grade point scale in Mexico is from 0 to 10, where 10 is the best grade; also, a grade of 6 is the minimum required to approve a course in the university where the study was performed. Questions included were about the time of wake up, if breakfast was consumed, the usual time of the day for this meal, and the subjective effect that skipping the meal had on their daily performance. Schedule of applying questionnaires and obtaining all information was between 7:00 am and 10:00 am.

Furthermore, we adapted and used the questionnaires first created by Pinto [26] and Fernández [27]. This questionnaire was adapted by assigning a score to each group of foods, according to the Mexican System Foods; finally, it was later validated obtaining a value of Cronbach’s alpha = 0.85.

Our questionnaire focused on breakfast nutritional quality in order to know the food groups that participants consumed. According to the Mexican Food System [28], a score was assigned for each serving of the following foods: 2 points for fruits (one medium-size or one cup of fresh fruit), vegetables (one cup raw, ½ cup cooked), animal protein sources (30 g of sausage, egg, chicken or beef, cheese, and others), and non-fat grains (one corn or wheat tortilla piece, a slice of bread, four cookies, 1/3 cup of oatmeal, potato, or amaranth); one point for dairy products (250 mL of milk or yogurt), fat-rich grains (1/3 of traditional Mexican sweet bread or tamale, fat-rich cookies), fats with protein (1/4 of an avocado, one spoon of chia, almonds, walnuts, or peanuts), non-protein fats (1/2 teaspoon of butter or vegetal oil); and 0 points for non-fat sugars (one teaspoon of jam, *cajeta*, or sweetened condensed milk); non-calorie foods (coffee or tea without nutritive sweeteners). Nutritional quality of breakfast was classified according to the sum of points of each serving of food that was consumed: Good quality (≥6 points), regular quality (3 to 5 points), and poor quality (≤3 points). These cut-off points were set based on the score of tertiles. The higher the score, the better variety and nutritional quality of breakfast.

#### 2.1.2. Cognitive Interference Assessment

From the several versions of the Stroop test, we chose the “Neuropsi Attention and Memory” [21], this has three sheets with 36 items each, distributed in four columns of nine items each. 

Sheet 1- *Words to be read*: Includes the words red, blue, green, and brown in a random order and printed in black ink. 

Sheet 2- *Color naming*: Consists of 36 color ovals in random order, different from the order of words in Sheet 1.

Sheet 3- *Interference*: Words in the first sheet are printed in the colors of the second sheet. In any case, the printed color is not the same as the meaning of the word.

Trained researchers administered the Stroop test to each participant individually. The time used to answer each test was recorded with a timer, and the errors were written down, according to Ostrosky-Solís et al. conditions of applications of the Stroop test [21]. The subject’s task in the three sheets was to read the words, name the colors, and say the ink color of the written word out loud, respectively, as fast as possible. For each sheet, the execution time was recorded as well as the number of correct answers. If the subject corrected a previously given answer, the assigned score was only for the first answer. Execution time for each sheet was classified according to the sample tertiles and the correct answers were distributed into two groups based on the median. 

Overall, the performance on the Stroop test was classified as good (36 correct answers with time on the first tertile), regular (36 correct answers but the time was on the 2nd or 3rd tertile, or with 35 or less correct answers and time was in the first tertile), and poor (correct answers were below 35 and the time was in the 3rd tertile).

#### 2.1.3. Anthropometric Assessment

Trained personnel used the Lohman technique [29] for weight assessment, with a scale (160 kg maximum capacity and a 0.100 kg precision). Height was registered with a stadiometer (0.1 precision range). With this information the BMI was calculated by dividing the weight in kilograms by the height in squared meters (weight/height^2^) and then categorized according to the World Health Organization criteria: Underweight (≤18.49), normal weight (18.50–24.99), overweight (25–29.99), and obese (≥30) [30].

#### 2.1.4. Statistical Analysis

Results are presented in frequencies and percentages for categorical variables, and measures of central tendency and measures of dispersion were obtained for continuous variables. Mean ± standard deviation was calculated when normality existed, and median and interquartile range were used when there was not. Normality was determined by Kolmogorov-Smirnov test when *p* > 0.05.

One-way ANOVA and post hoc tests were used for means comparison, and the Pearson Chi^2^ test was used to assess the association between breakfast nutritional quality and cognitive interference categories. The cognitive interference values were divided into two categories to identify the variables related to poor performance. Food groups were assessed as a two-category variable to calculate the odds ratio for food group omission. Logistic regression models were performed to identify breakfast variables associated with poor performance on cognitive interference test.

## 3. Results

As shown in Table 1, 422 students participated; 238 (56%) were females and the rest were males. The median age was 22 years-old, the mean BMI was 23.5 ± 4, and mean daily hours of sleep was 6.4 ± 1.3.

We found that 17 (4%) of the university students had underweight, 282 (67%) had normal weight, 87 (21%) were overweight, and 36 (9%) were obese. Regular breakfast consumption was reported in 292 (69%) participants. A total of 344 (81.5%) students considered that skipping breakfast has an adverse effect on physical and cognitive performance. The 71% (*n* = 299) of the participants said that the food choice during the rest of the day is different when skipping breakfast. For instance, 161 (54.1%) of the participants who skip breakfast, ate more energy-dense foods, and 138 (46%) ate more servings of the available food. Fifty-one percent of all participants referred that skipping breakfast could predispose them to eat a lot more during the rest of the day.

Table 2 shows that when breakfast was consumed after one hour of waking up, there was an increased likelihood of not eating any fruits; at this time more fat-rich grains were consumed.

Execution time and correct answers in the Stroop test in relation to breakfast nutritional quality is shown in Table 3. The number of correct answers was similar for the three sheets regardless of breakfast nutritional quality; nevertheless, execution time was longer for those with a poor breakfast.

The analysis of Stroop test results according to breakfast nutritional quality showed that those with good nutritional quality breakfast presented a regular to good performance; while a regular and poor breakfast was also more frequent in those classified as regular and poor performance (Table 4). There was no association between gender and cognitive interference (*p* = 0.080).

Table 5 shows that the variables related to poor performance in the Stroop Test were: For word reading, the lack of non-fat grains and vegetables; for color naming, lack of fruits and food of animal protein sources; on the other hand, fat-rich grains was a protective factor for this kind of task. Poor interference, which means reduced ability to inhibit interference, increased when they did not eat vegetables, non-fat grains, and with a poor nutritional quality breakfast in general. Lack of dairy products was not related to poor performance.

## 4. Discussion

The results of this study show that a poor nutritional quality breakfast may have an adverse effect on cognitive interference performance in university students. Furthermore, we found that a poor nutritional quality breakfast increases the likelihood of consuming low nutritional quality foods in the following meals of the day. This behavior may contribute to weight gain and increase metabolic risk [8,10,31].

### 4.1. Participants and Breakfast Characteristics

Regarding the nutritional status in our sample, 30% of the participants were overweight and obese. This figure differs from the 72.5% reported for Mexican adults (>20 years), described in the 2016 Halfway National Health and Nutrition Survey [32]. The reason for this difference could be that in our study we only assessed young adults (18–25 years) and the prevalence reported in ENSANUT MC 2016 is for adults older than 20 years.

In our study, participants who eat breakfast after one hour of waking up eat less fruits and more fat-rich grains. Similarly to our results, Australian adults who skip breakfast were less likely to eat grain foods, fruits, and vegetables; and were more likely to eat refined foods, compared to breakfast consumers [33]. Also, breakfast skippers had the highest intake of added and free sugars, and the lowest micronutrient intake [34]. Nevertheless, a study performed in obese adults showed that those with extended morning fasting did not have compensatory intake during lunch nor did it increase appetite during the afternoon.

On the other hand, about half of our sample consumed breakfast. It is reported that eating some foods, even if it is something of low nutritional quality, is better than skipping breakfast. The explanation may be because the energy expenditure is low during the morning when remaining fasted compared to having had some food for breakfast [35,36].

Our research showed that about half of the university students who skip breakfast eat bigger servings in the following meal time. Overall, about half of the participants said that when skipping breakfast, they chose to eat more energy-dense foods in the rest of the day. Regarding the above mentioned, it has been documented that breakfast omission increases hunger and the consumption of bigger servings in subsequent meals [37].

### 4.2. Nutritional Quality Breakfast and Cognitive Interference

Our results show that the time required for word reading and interference was shorter for those that had a good nutritional quality breakfast. Similar findings were observed in children and adolescents; those who ate breakfast had better outcomes on concentration tasks [24]. This outcome could be explained by the supplying of glucose into the brain after eating breakfast, as has been reported by Nilsson et al. [38].

In our study, we did not observe an association between nutritional quality of breakfast and cognitive interference. However, differences existed regarding the time to answer. We did not find scientific evidence to compare our results about the time to answer the test.

The nutritional quality of breakfast or its composition has not a clear consequence for cognitive interference in other age groups [39]. In our study, those participants classified with a regular or poor nutritional quality breakfast had also more frequency of regular and poor cognitive interference compared to the participants with a good nutritional quality. Therefore, our hypothesis was not supported; which probably could be explained by the variability between the timing of the administration of the Stroop test and the time after having breakfast [40,41,42,43]. Our participants responded to the test between 0 and 6.5 hours after having breakfast, even though we had an established schedule to collect data, the participants had a different number of hours after having had breakfast. Therefore, this may be one of the many arguments of the inconsistency to draw any conclusion, as other studies reported it [24,43]. In addition, there are other factors that may influence the cognitive interference performance, such as exercise and stress [44,45] which we unfortunately did not assess.

### 4.3. Importance of Type of Food and Food Composition in Cognitive Interference

In our study, a good performance on the cognitive interference test was observed in participants who had eaten carbohydrates at breakfast, such as vegetables, fruits, and non-fat grains. Other studies [39,43] reported that having an adequate breakfast has an effect on executive functions, for example, Edefonti et al. showed that for children and adolescents, breakfast had an effect on memory and attention; furthermore, they identified that food with a low glycemic load is related to a better performance on cognitive tasks [24].

We observed a low consumption of vegetables in breakfast for all participants; this is also reported for other age groups and for every meal [46,47]. In Mexico, 28.4% of the women and about 20% of children consume the recommended intake of vegetables [48,49]. For the general Mexican population, only 6% of the total energy intake comes from fruits and vegetables [50]. Unfortunately, one form of consuming vegetables is in juice mixed with fruits during breakfast [51]. The vegetable intake is insufficient in young adults; this pattern could continue later in life and even maybe pass on to the next generations through family eating habits [51,52]. Thus, it is recommended to promote a consumption of wider variety of food, together with other lifestyle factors [52], to maintain health. 

### 4.4. Strengths and Limitations

Our study demonstrated that the nutritional quality of breakfast was associated with the time spent to answer the Stroop test; there is little evidence about this association. Regarding the limitations of this study, there could be other factors contributing to deficient interference scores, as the level of academic stress, the family food environment from previous life stages [53] or even the glycemic index [54]. Also, because of the cross-sectional design, causality cannot be assumed in the associations that we found.

For future studies, it is recommended to include the assessment of physical activity [55,56,57] and quality of sleep [58,59].

## 5. Conclusions

In our study performed in a sample of university students, the nutritional quality of breakfast was associated with the time spent answering the Stroop test, but not with cognitive interference.

## Figures and Tables

**Table 1:** General characteristics of the participants, *n* = 422.

Variable	Mean ± Standard Deviation	Min–Max ^d^
Height (cm)	166 ± 8	150–193
Age (yrs) ^a^	22 (20–25)	18–25
Weight (kg) ^a^	65.5 (56–72)	43-116
BMI (kg/m^2^)	23.5 ± 4	14–34
Grade point average	8.8 ± 0.6	7–10
Daily sleep hours (h)	6.4 ± 1.3	3–10
Time of breakfast ^a^	7:00 (6:00–9:00)	4:50–11:50
Time between waking-up and breakfast (h) ^a^	1 (0.5–3.0)	0.0–6.5

^a^ Data in median (percentile 25–percentile 75). ^d^ Minimum and maximum values.

**Table 2:** Risk to omit food groups according to the time from wake up to having breakfast.

Time from Wake Up to Having Breakfast.					
Omitted Foods Groups	>1 Hour *n* = 185	≤1 Hour *n* = 237	OR	95% CI	*p*-Value
	*n* (%)	*n* (%)			
Fruits	76 (41)	74 (31)	1.53	1.02, 2.29	0.036
Vegetables	139 (75)	168 (71)	1.24	0.80, 1.91	0.331
Animal protein sources	78 (42)	78 (33)	1.48	0.99, 2.21	0.051
Dairy products	74(40)	76 (32)	1.41	0.94, 2.10	0.091
Non-fat grains	62 (34)	60 (25)	1.48	0.97, 2.27	0.065
Fat-rich grains	107 (58)	171 (72)	0.52	0.35, 0.79	0.002

OR: odds ratio, CI: Confidence Interval.

**Table 3:** Execution time and correct answers in the Stroop test in relation to breakfast nutritional quality.

		Breakfast Nutritional Quality			
Stroop Performance	Total	Good *n* = 282	Regular *n* = 79	Poor *n* = 61	*p-*Value
Time (seconds)					
Word reading ^£^	15.2 ± 2	14.2 ± 2 ^§¥^	15.5 ± 2 ^£^	16.1 ± 3	0.001
Color naming ^b^	19.5 ± 3	19.1 ± 3	19.8 ± 2 ^£^	20.3 ± 3	0.051
Interference ^c^	29.7 ± 6	29.3 ± 6 ^§¥^	30.4 ± 6 ^£^	32.9 ± 6	0.006
Correct answers					
Word reading ^a^	35.9 ± 0	35.9 ± 0	35.9 ± 0	35.9 ± 0	0.757
Color naming ^b^	35.7 ± 1	35.4 ± 1	35.1 ± 1	35.3 ± 2	0.106
Interference ^c^	34.6 ± 1	34.6 ± 2	34.2 ± 2	34.6 ± 1	0.327

^a^ Sheet 1, ^b^ Sheet 2, ^c^ Sheet 3. Data in means (± standard deviation). p-value obtained with ANOVA test. Post-hoc test: *p* < 0.050 ^§^ Good vs. Regular ^¥^ Good vs. Poor ^£^ Regular vs. Poor.

**Table 4:** Breakfast nutritional quality and cognitive interference performance.

	Breakfast Nutritional Quality			
	Good (*n* = 282)	Regular (*n* = 79)	Poor (*n* = 61)	*p*-value
Interference performance				
Good (*n* = 124)	86 (30)	26 (33)	12 (20)	0.001
Regular (*n* = 184)	132 (47)	35 (44)	17 (28)	
Poor (*n* = 114)	64 (23)	18 (23)	32 (53)	

Data expressed in frequencies of cases (%). *p*-value obtained with Pearson Chi^2^ test.

**Table 5:** Association between poor performance on cognitive interference and the omission of breakfast.

	OR	Standard Error	95% CI	*p*-value
	Word Reading			
No vegetables	2.78	1.48	0.93, 2.35	0.095
No non-fat grains	1.70	0.25	1.03, 2.81	0.037
Constant	1.58	0.19		0.022
	Color Naming			
No fat-rich grains	0.55	0.21	0.36, 0.85	0.008
No fruits	1.55	0.21	1.43, 1.99	0.049
No animal protein sources	1.63	0.21	1.07, 2.49	0.022
Constant	2.11	0.20		0.001
	Interference			
No vegetables	2.72	0.29	1.25, 4.80	0.001
No non-fat grains	2.65	0.26	1.28, 4.68	0.007
No dairy products	1.65	0.27	0.96, 2.83	0.066
Poor breakfast	1.51	0.33	1.51, 1.97	0.043
Constant	1.48	0.29		0.014

Logistic regression model adjusted for BMI, gender, grade point average, and sleep hours.
